# Developing an analytical framework for assessing progress toward ecosystem-based management

**DOI:** 10.1007/s13280-015-0655-7

**Published:** 2015-05-28

**Authors:** Sara Borgström, Örjan Bodin, Annica Sandström, Beatrice Crona

**Affiliations:** Stockholm Resilience Centre, Stockholm University, Kräftriket 2B, 10691 Stockholm, Sweden; Luleå University of Technology, 971 87 Luleå, Sweden

**Keywords:** Ecosystem-based management, Policy cycle, Social–ecological system, Management process, Evaluation

## Abstract

Ecosystem-based management (EBM) has become a key instrument of contemporary environmental policy and practice. Given the increasingly important role of EBM, there is an urgent need for improved analytical approaches to assess if and to what extent EBM has been accomplished in any given case. Drawing on the vast literature on EBM, we identify five key ecosystem aspects for assessment. By linking these aspects to four phases of management, we develop an interdisciplinary, analytical framework that enables a high-resolution and systematic assessment of the degree of specificity and integration of ecosystem aspects in an EBM. We then apply the framework to evaluate five coastal EBM initiatives in Sweden, four on the Baltic coast and one on the west coast. Our results demonstrate our framework’s usefulness for in-depth and continuous assessments of processes aiming for EBM, and also provide an empirical basis for inferences about the key challenges for successful EBM.

**Electronic supplementary material:**

The online version of this article (doi:10.1007/s13280-015-0655-7) contains supplementary material, which is available to authorized users.

## Introduction

Ecosystem-based management (EBM) can be seen as an overarching strategy to handle the complexity of environmental challenges, which has been developed from research into policy and practices since the 1990s. Today EBM is recognised at a global policy level through e.g., the Malawi principles, which guide the implementation of the Convention on Biological Diversity (UNEP 1998; CBD 2014) (Supplementary Material S1a; Supplementary Material is hereafter referred to as SM). It has also become one of the main guiding principles in environmental governance at national, regional, and local levels, such as Integrated Coastal Zone Management, ICZM (e.g., Belfiore 2003) and several European Union policies (Apitz et al. 2006), e.g., the Water Framework Directive (European Commission 2000), the Marine Strategy Framework Directive (European Council 2008), and the Landscape convention (Council of Europe 2000).

While both academic and gray literature on EBM and its constituents has burgeoned in recent decades, there is still a lack of systematic, critical appraisal of EBM progress and outcomes that take both ecological and socioeconomic aspects into account in an integrated fashion. This is problematic given the increasingly important role of EBM as a guiding principle and goal in both policy and practice (Pirot et al. 2000; Hartje et al. 2003; Smith and Maltby 2003). A possible reason for this lack of outcome assessment is the lack of clear and specific definitions, valid across disciplinary boundaries (cf. Tallis et al. 2010). Broadly speaking, EBM seeks to adapt planning and management to the dynamics of whole ecosystems; however, the interpretations of what this means vary greatly. Such variation in practice makes comparison of EBM progress across cases difficult.

This article takes a first step in addressing these issues by developing an analytical framework that gives both a broad overview and a high-resolution, systematic assessment of the degree to which EBM can be said to have been achieved. Accomplishing EBM requires substantial time and effort, thus one can expect that many attempts do not reach full-fledged EBM. Therefore, the implementation of EBM should be seen and evaluated as a process. Our analytical framework is generic and applicable to any kind of environment, but is here illustrated by coastal system examples. By evaluating the recognition of different ecosystem aspects across multiple management phases we enable assessment of the degree of system thinking, the degree of specificity and the degree of integration throughout the EBM process. Furthermore, dividing EBM into sub-components, as we do in our framework, provides for an evaluation of which aspects of the EBM approach create the most difficulties across contexts.

The development of the EBM approach sprung from a recognition among biology, ecology, and environmental management scholars that, as part of a more ecosystem-oriented approach to resource management, there was a need to consider social aspects such as power relationships and human values in management designs (e.g., Slocombe 1993, 1998a; Grumbine 1994; Christensen et al. 1996). To date, two development pathways of EBM research can be identified. One focusing on the social and institutional processes linked to EBM, where social factors for success, such as degree of participation of various stakeholder groups, and diversity of knowledge and trust among actors involved in management are emphasized (Cortner et al. 1998; Imperial 1999; Bissix and Rees 2001). This is also reflected in many of the currently adopted key policy documents (Shepherd 2004; CBD 2014). The other pathway focuses on the ecological aspects, assessing current ecological status and ecological outcomes of EBM in specific environments or related to certain policy frameworks (Borja et al. 2008), e.g., Water Framework Directive (European Commission 2000) and European Marine Strategy Directive (Fletcher 2007; European Council 2008), often with limited attention to the social processes of EBM. Hence, the linkages between ecological and social aspects in EBM are still underdeveloped. By linking the ecosystem aspects to specific phases of the management cycle we attempt to create an analytical assessment tool that simultaneously assesses ecological goals and ambitions, as well as social processes, management strategies, and actions.

The paper first outlines the methodological approach, discussing the components of the EBM, as well as the details of the assessment framework. Next we provide a detailed description of how the framework was applied in one case, then we add four less detailed cases and a comparison between them. We conclude by discussing the key contributions of the assessment framework, its limitations as well as the broader insights it can provide, regarding the EBM approach in general and its progress in practice.

## Developing an analytical framework for assessing EBM processes

### Addressing multiple ecosystem aspects and management phases

Deciding which ecosystem aspects to include when assessing the degree of EBM is not trivial. How should the system be delimited for management, what are the relevant spatial and temporal scales for major ecological processes and what is an appropriate balance between natural resource use and biodiversity conservation? By combining a seminal work on the scientific foundation for EBM (Christensen et al. 1996) with the Malawi principles that directly address ecology (SM-S1a), and also drawing on key ecologically focused EBM publications (Slocombe 1993, 1998a, b; Sexton 1998; Yaffee 1999; Crowder and Norse 2008) and the IUCN EBM guide (Pirot et al. 2000), we identify five ecosystem aspects of EBM: (1) Biodiversity (genetic, species and biotopes), (2) Relations and Ecological Processes, (3) Changes and Uncertainty, (4) Scales (temporal and spatial), and (5) Anthropogenic Processes. The scientific rationale and detailed content of each of these five ecosystem aspects is given in SM-S1b.

**Fig. 1:**
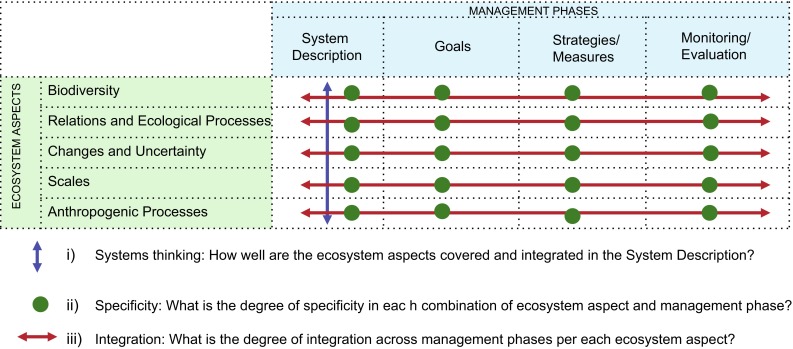
The EBM assessment matrix combines a set of ecosystem aspects with management phases derived from ecological and political science literature. It allows for a high-resolution assessment of a variety of data concerning an ecosystem-based management as well as triangulation of different kinds of data. After the initial, systematic sorting of data the matrix can be used for multiple, both detailed and more comprehensive, analyses. In this study, five coastal EBM were assessed regarding the degree of systems thinking, degree of specificity of ecosystem aspects and management phases and degree of integration between and across aspects and phases

While the above-mentioned ecosystem aspects constitute, as we argue, the foundation of EBM and assessments thereof (rows in Fig. 1), it is important to note that the implementation of EBM is often gradual, with several different management phases in which different tasks are executed, and span long time periods in an on-going management cycle (cf. de Leon 1999). There is, therefore, a need for a process-oriented approach when evaluating EBM, that disentangles how the ecosystem aspects are handled in different management phases (dealing with different tasks): in the definition and description of the social–ecological system, in the formulation of management goals, in the selection of measures to achieve these goals, as well as in the indicators for monitoring. For the purpose of our analysis, we denote these four phases: System Description, Goals, Strategies/Measures, and Monitoring/Evaluation (columns in Fig. 1). Hence, assessing the success of an EBM process is not just a matter of evaluating the final outcomes, or merely one of its phases, but must encompass all phases as well as an analysis of how they interconnect (e.g., Olsen 2003; Pickaver et al. 2004).

Similar categorization of management phases or stages has been applied in the study of policy processes (Parsons 1995; de Leon 1999) and in adaptive management research (cf. Pickaver et al. 2004; Plummer 2009). The notion of stages can, however, be criticized for simplifying and rationalizing processes that are inherently disorderly structured, iterative and dynamic in character [compare with the critique toward the stage-heuristic model in policy analysis (Carlsson 2000b)]. However, distinguishing key stages in the management process allows us to break it down into activity-related steps that can be analyzed and assessed. Yet, we consider them as mere heuristic devises that help in structuring the empirical data for analysis and conceptualize them more as functions of management rather than as predestined phases following a specific order (cf. Carlsson 2000a).

System Description refers to the functions of knowledge generation and assessments of the ecosystem; general and specific characteristics, history, functions and appreciated values, present status, perceived negative impacts and projected future changes. During this phase, management is targeted at describing the characteristics of the social–ecological system. Goals involve the prioritisation of values setting overall management goals as well as detailed directions of management. This second phase relates to the important task of defining management goals, which is followed by the Measures/Strategies phase that includes management practises, strategies, and actions necessary for reaching the goals. Finally, every management process in an EBM context should consider monitoring and evaluation. The Monitoring/Evaluation phase includes the construction of evaluation criteria for how to monitor the system and the possible impact of different management strategies. Monitoring and evaluation is essential to help management close feedback loops between the ecological dynamics and the management design, to allow for prompt adjustments of management to changes in the system, i.e., the adaptive capacity (Folke et al. 2005). With the point of departure in these phases, that are distinct in the way that they address different tasks and organizing functions, we assess to what extent, and how, the ecological aspects are considered in management (columns in Fig. 1).

### The EBM assessment matrix

The EBM assessment is organised into three steps; (1) collecting and filling the assessment matrix comprising both the management phases as well as the key ecosystem aspects (Fig. 1) with information, (2) analyzing the matrix content using three guiding questions, and (3) developing contextualized examples (based on our generic criteria described below) and use these to score to the overall progress of management toward EBM.

The matrix in Fig. 1 organises the information about the EBM under evaluation, collected from written records (plans, reports, meeting notes), interviews (with decision makers, stakeholder and/or managers, etc.) or combinations of information sources. The first step reveals how different ecosystem aspects are addressed in different phases of the management cycle. Practically, this step could be conducted using some off-the-shelf methods for structuring data in adequate ways (e.g., coding of qualitative data). The second step of analysis is to evaluate the matrix content in terms of the degree to which it fulfills EBM, and thereby also reveal strengths and weaknesses, gaps and linkages in present management, of relevance for improvements.

We used three overarching questions specifically aimed to capture the very essence of EBM (systems thinking, specificity, and integration, Fig. 1) to analyze the matrix content. First, in order for EBM to succeed it is essential to have a comprehensive understanding of the system to be managed. The management phase Systems Description must therefore include descriptions of all ecosystem aspects including their interrelations. Thus our first question aims to capture this degree of systems thinking by asking: How well are the ecosystem aspects covered and integrated in the System Description (first column in Fig. 1)? Another challenge in reaching EBM is to be clear about what is to be managed under each of the different ecosystem aspects and how. This is fundamentally an issue of specificity and thus our second analytical question used to interrogate the matrix is: What is the degree of content specificity in all ecosystem aspects and management phases? This question is used to evaluate the details of each of the matrix cells (Fig. 1).

While having a good and integrated system understanding elaborated under System Description and a high degree of content specificity throughout the matrix are essential preconditions, it is not sufficient to reach EBM. This detailed understanding must inform the whole management cycle (including all its phases), meaning that the System Description must be matched by the content and specificity across the management phases in a coherent and integrated way. This is a way of assessing integration of the individual ecosystem aspects across management phases, and is captured through the question: What is the degree of integration across management phases per ecosystem aspect? (rows in Fig. 1). In the final step, scores of ‘high’, ‘medium’ or ‘low’ are given for systems thinking, specificity, and integration. We have developed criteria for the different scores that are generic and intended to be applicable across cases (Table 1). However, to be useful these general criteria must be related to the specific context of the evaluated EBM process. These specifications could be derived from an external expert committee, from relevant literature, from the actors in the particular EBM, or any combination of these (Turnhout et al. 2007; Tengö et al. 2014). In Table 1 the contextualised criteria we used when evaluating the plans of the studied coastal areas are presented.

**Table 1:** Evaluation scheme. Generic criteria and contextualised criteria specifying the scores used for evaluating the management progress toward EBM by the three overarching, analytical questions

Component	Score	Generic criteria	Contextualised criteria
*Systems thinking* How well the ecosystem aspects are covered and integrated in the System Description (first column in Fig. 1)	High	All ecosystem aspects and several linkages between them are addressed in detail	Biodiversity is addressed at genetic, species and landscape levels, different relations in the system (e.g., food webs) are described as well as essential processes (e.g., nutrient cycling, hydrology);, spatial and temporal scales are discussed in relation to biodiversity, relations and processes. Historical and future projected changes of the system are discussed, as are key uncertainties in the system. Many anthropogenic processes are identified and related to the system dynamics
	Medium	Most ecosystem aspects and some linkages are addressed, but varying degrees of detail	One level of biodiversity is described, with the others just mentioned. Relations and ecological processes are mentioned in general terms and related to biotopes, with spatially and temporally explicit information for some species. Change and uncertainty are mentioned but in general terms (e.g., a general lack of knowledge about populations). Many anthropogenic processes are addressed in detail but without clear connections to the ecosystem dynamics (e.g., touriSM—forestry, recreation, pollution and transportation are presented regarding history, future prognosis and as problematic to other values in the system)
	Low	A few ecosystem aspects and linkages between them are addressed in general terms	Only one level of biodiversity is described (e.g., landscape) and relations are not addressed. Some ecological processes are mentioned in general terms (e.g., importance of water fluctuations for fish spawning). There is no recognition of spatial or temporal scales. Change is mentioned in the introduction (e.g., importance of climate change) but not further addressed. Several anthropogenic processes are addressed generally, but not related to the ecosystem dynamics
*Specificity* The degree of content specificity in each combination of ecosystem aspect and management phase (each cell in Fig. 1).	High	The degree of specificity is ‘high’ for most ecosystem aspects and management phases	In the system description the ecosystem aspects are described in detail (e.g., quantities, history, future trends, spatially explicit). There are overarching goals, as well as interim targets that are spatially and temporally explicit (e.g., sustainable fish populations, including defined populations goals per species and at certain times and places). The measures are specific in what, where and when and in terms of expected outcomes. There is a monitoring program that follows the system in detail (e.g., changes in populations over time, nutrient leakage from certain sources)
	Medium	The degree of specificity varies across ecosystem aspects and management phases	Most ecosystem aspects are presented in detail in the system description. Some goals are quantitative (e.g., level of nitrogen), others general (e.g., sustainable forestry). The measures are detailed, who is going to do what, when and where. The monitoring varies greatly in specificity, from high, e.g., the level of nitrogen at several points over time to low, e.g., no specific monitoring for the system aspects
	Low	The degree of specificity is low for most ecosystem aspects and management phases	EAs and management phases are mostly very generally formulated or there is a strong focus on a single ecosystem aspect and/or management phase. E.g., there is a general focus on nutrient cycling and eutrophication that is described in detail (history, future prognosis, spatial variations, anthropogenic sources, ecological consequences). The goal is to decrease nutrient leakage in certain places at certain times and a variety of measures are presented that are to be followed up by targeted monitoring
*Integration* The degree of integration across management phases per ecosystem aspect (each row in Fig. 1)	High	Content and specificity are matched across all management phases for each ecosystem aspect	All levels of the ecosystem aspect biodiversity addressed in the management phase system description are targeted by goals at the same level of specificity. The goals are related to measures that are linked to the targeted biodiversity components. Finally there is a monitoring scheme that follows the progress toward the goals and the effectiveness of the measures over time
	Medium	The content and specificity are somewhat matched across some management phases for some ecosystem aspect	For example even if the ecosystem aspect processes are generally described, there are detailed and quantitative goals related to processes. However measures to reach these are just vaguely formulated, whereas the monitoring of the goals is rigorous
	Low	The content and specificity are not matched across all management phases per ecosystem aspect	The content and specificity in how for example ecosystem aspect anthropogenic processes are described are not matched in the following management phases; the goals are general, the relation to measures unclear and there is no monitoring related to anthropogenic processes

### Case study application

**Fig. 2:**
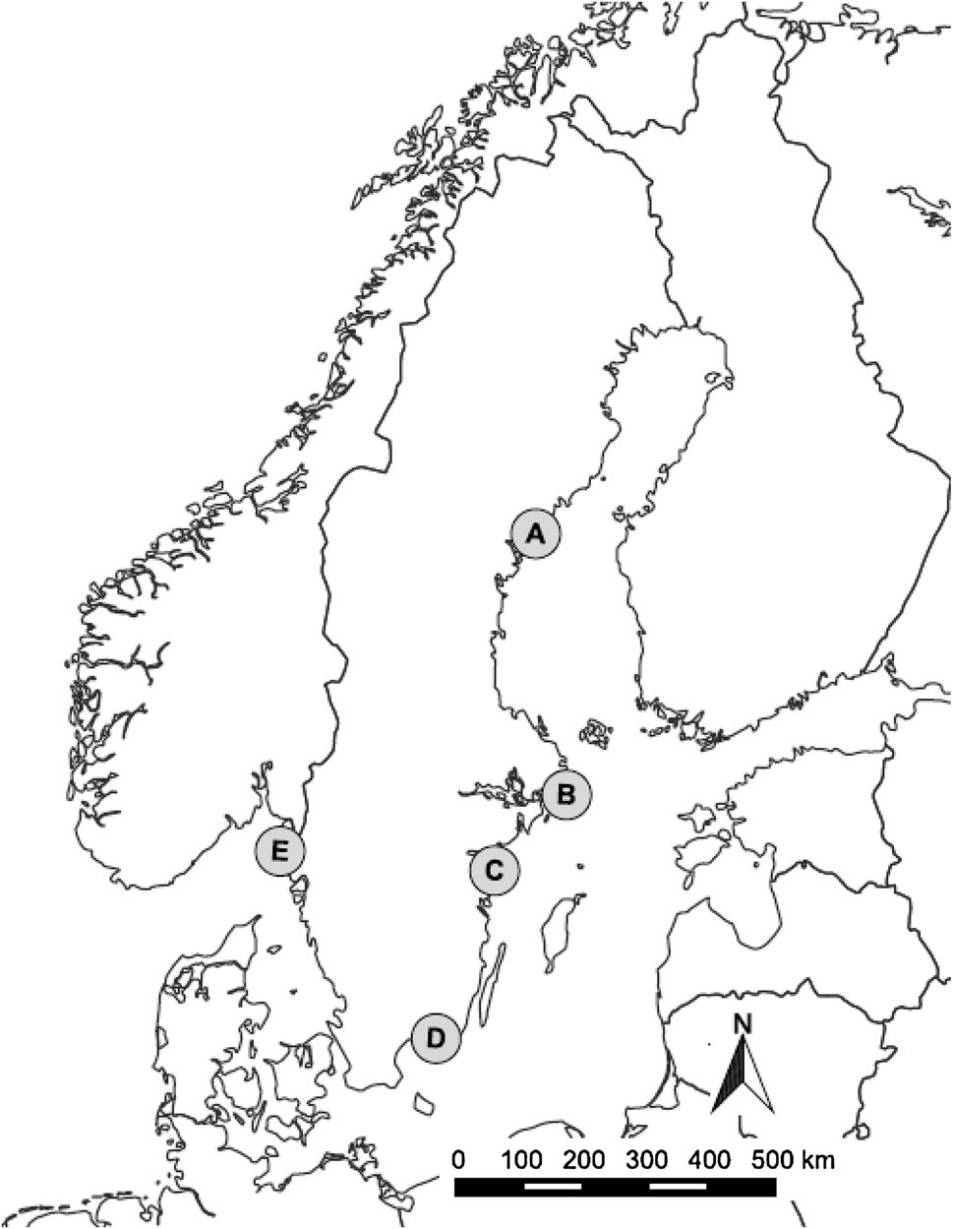
The coastal areas included in the case study; *A* Höga Kusten (HK), *B* Stora Nassa–Svenska Högarna (SNS), *C* St Anna–Missjö (SAM), *D* Blekinge Archipelago (BA), *E* Norra Bohuslän (NB). For further details see SM-S2

Five coastal areas in Sweden were used to test the assessment framework (Fig. 2, SM-S2). For these areas the Swedish Environmental Protection Agency (SEPA) initiated planning processes framed by the EBM concept including a strong focus both on integrated coastal zone management (cf. Olsen 2003) and stakeholder participation (for details, see Sandström et al. 2014). The SEPA recommended certain working procedures to be applied in all five coastal areas for information collection, designating ecologically relevant themes, and stimulating stakeholder participation. All areas formulated their own management plan and these plans were well suited for a comparative study, since the differences in outcomes among the five areas were paired with similarities in instructions and framework provided by SEPA.^1^^,^^2^

The assessment framework aims to be applicable to whole management processes where this kind of development of a plan is just one phase among others in the management process. However, the plans describe management intentions across all management phases and hence constitute relevant, useful, and effective material on which to test the assessment framework. Atlas.ti software^3^ was used for the qualitative content analysis (Friese 2012) and a set of codes was derived from the categories in the matrix (Fig. 1) and excerpts from the plans were then extracted by each code and combinations of codes, following the matrix structure and analytical questions. The coded material was condensed and filled into one matrix per plan (SM-S3). The same material could occur in several cells in the matrix, if considered relevant across management phases and/or ecosystem aspects (i.e., the material was covered by several of the codes). In the following we summarily present the EBM assessment of one of the areas, St Anna Missjö (SAM area, “C” in Fig. 2) and a comparative analysis of the assessment of all five areas. A detailed report of the SAM area assessment is found in SM-S4. The assessment matrix and score tables for all five areas are found in SM-S3 and S5.

## Results

### Summary of the EBM assessment of the SAM area plan

The SAM area management plan process followed the procedure suggested by Open standard^4^ with formulation of a vision, identification of preservation values, goals and an analysis of impact factors and drivers of those (see SM-S3 for assessment matrix and SM-S4 for in depth analysis).

In the System Description the specificity was ‘medium’ to ‘high’ regarding ecosystem aspects Biodiversity and Relations and Ecological Processes, indicating that the plan presents a rather comprehensive view of the basic components of the system (Table 2; first column in SM-S3a). However, the ecosystem aspects—Changes and Uncertainty, and Scales—were formulated in more general terms. For example, some ecosystem aspects were presented on spatially explicit maps, but these were not overlaid for a more integrated analysis of the interaction between ecosystem aspects. No other uncertainties were identified than knowledge gaps. Least details in the System Description were found for the ecosystem aspects, Changes and Uncertainty, and Scales. In summary, the SAM area plan System Description addressed all ecosystem aspects but with a differing degree of detail and the content was not fully integrated between the ecosystem aspects. This gave the score ‘medium’ for systems thinking.

**Table 2:**
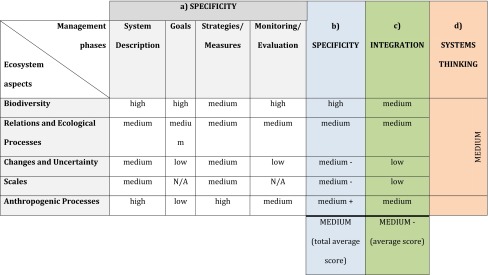
Summary of EBM assessment in the SAM area (C in Fig. 2). (a) Scoring of the specificity in each combination of ecosystem aspect and plan phase (*white cells*), (b) Average specificity score per ecosystem aspect across management phases (*blue cells*), (c) Scoring of integration per ecosystem aspect (*green cells*), (d) Aggregated score of the degree of systems thinking (*orange cells*). For specification of each assessment criteria see Table 1. N/A indicates that this aspect was not reported in data

For most of the combinations of ecosystem aspects and management phase the evaluated specificity was ‘medium’. Least specificity was seen for the ecosystem aspect Scales in the management phases Goals and Monitoring/Evaluation, since these were missing in the plan. Except for the ecosystem aspect Biodiversity, the management phase Goals was rather vague and general, e.g., formulated in terms of “sustainable boating” and “low degree of exploitation”. Generally, the less developed the ecosystem aspect in the System Description, the more general was the Goals phase. For example, the descriptions concerning Relations and Ecological processes were less specified than for Biodiversity and in the management phase Goals rather relative.

The degree of integration across management phase per ecosystem aspect was ‘medium’ to ‘low’ in the SAM area plan (rows in SM-S3a). For ecosystem aspect Biodiversity there was matching between System Description, Goals and Monitoring/Evaluation in terms of specificity and content, whereas the Strategies/Measures were much more generally formulated and hence created gaps between management phases. Since just some relations were addressed in the Goals there was not a complete match between the content and specificity in System Description and Goals regarding ecosystem aspect Relations and Ecological Processes. For ecosystem aspect Relations and Ecological Processes many of the existing goals were rather general and relative. The suggestions in the Strategies/Measures phase did not match the specificity in the System Description concerning ecosystem aspects Relations and Ecological Processes. The trends and prognosis formulated in the System Description were not matched in the other management phases. Even though an overall adaptive approach was highlighted in the introduction of the plan, as well as the necessity of evaluation, this was not further specified and a monitoring program was not presented. Since the monitoring program is missing, the adaptive approach of the plan becomes unfeasible. Generally, the ecosystem aspect Scales were addressed only in the System Description and hardly in the other management phases. Hence, there was a severe lack of integration of the ecosystem aspect Scales across management phases. The goals concerning Anthropogenic Processes were very general, e.g., “sustainable fishing and boating” and some were prohibiting, e.g., “no dredging”, while the suggestions in management phase Strategies/Measures were most rigorous and detailed for this aspect of the system. There were no indicators in Monitoring/Evaluation related to Anthropogenic Processes, since the presented indicators focused on the end result, not the outcomes of a certain activity. This limits the possibilities to adjust management to system changes. The plan suggested quantitative indicators for the preservation values as a way to specify the goals. However, there are no suggested indicators of the impact factors which are the main target of the suggestions under management phase Strategies/Measures. The sections given the most weight in the SAM area plan were the management phase System Description and the ecosystem aspects Biodiversity and Anthropogenic Processes.

### Outcomes and insights from the comparative application

A similar analysis as for the SAM area plan was made also for the other four plans (SM-S3). Here we present a comparative analysis of the material and discuss similarities and differences among the five areas (Table 3; for details see SM-S5).

**Table 3:** Summary of EBM assessment of the five case study areas. IF: Impact factor. For details see assessment and analysis matrices for each case study area separately in SM-S3 and S5

Area	Systems thinking	Average specificity	Average integration	Gaps and points of gravity
HK (A)	Medium	Medium	Medium−	All ecosystem aspects are included in the System Description but with varying specificity and linkages between aspects The highest specificity is found in Systems Description and Monitoring/Evaluation, but is not matched in Goals and Strategies/Measures phases, creating a gap in the management process Weak match across management phases, with the highest specificity in the System Description Anthropogenic Processes are well specified in Goals and Strategies/Measures but less so in System Description and Monitoring/Evaluation The monitoring system is described in detail, focusing on species and abiotic factors in terms of Relations and Ecological Processes by the use of indicators and the need for continuously evaluation and updating the management plan is addressed The main emphasis is on System Description and Monitoring/Evaluation of all ecosystem aspects except for Anthropogenic Processes which are the focus of Goals and Strategies/Measures
SNS (B)	Medium	Low+	Low	Strong focus and highest specificity at the species level of biodiversity across plan phases, terrestrial biotopes mentioned only in the Systems Description Mismatch between System Description and the other plan phases for Relations and Ecological Processes, and Scales, regarding both content and specificity. Scales not addressed in Strategies/Measures or Monitoring/Evaluation Change and Uncertainty is addressed across phases but with low specificity The main emphasis is on the System Description and ecosystem aspects Biodiversity and Anthropogenic Processes
SAM (C)	Medium	Medium	Medium−	The specificity and integration is ‘medium’ to ‘high’ for Biodiversity, Relations and Ecological Processes Change and Uncertainty, and Scales are formulated in general terms, indicating a lack of understanding of system dynamics Goals formulation are general and vague Severe lack of integration of Scales No other uncertainties other than knowledge gaps are identified No monitoring program, hence the adaptive approach is unfeasible Quantitative indicators for the PVs are suggested as a way to specify the goals, but no indicators are suggested for the IFs that are the main targets of the suggested measures The main emphasis is on System Description and the ecosystem aspects Biodiversity and Anthropogenic Processes
BA (D)	High	Medium	Low	All ecosystem aspects described in detail in a document from the Man and the Biosphere Area (MAB) designation process^a^ The specificity in the System Description is not matched in the other plan phases, except for Biodiversity and Anthropogenic Processes The monitoring suggested in the MAB-document is not linked to the Goals and Strategies/Measures in the plan, making the adaptive approach unfeasible System Description and Monitoring/Evaluation are not matched by the Goals and Strategies/Measures suggested, indicating a failure to operationalize the MAB process No uncertainties other than knowledge gaps are identified The main emphasis is on System Description and the ecosystem aspects Biodiversity and Anthropogenic Processes
NB (E)	High	Medium	Medium+	All ecosystem aspects are addressed with rather ‘high’ specificity in the System Description, including their linkages Weak match across phases for Scales, where the description is detailed, but scales are missing in Goals and Monitoring/Evaluation and only generally addressed in Strategies/Measures Monitoring/Evaluation is lacking for Change and Uncertainty, Scales and Anthropogenic Processes hampering integration across phases Measures focus on societal dynamics and are very specific and detailed, while the linkages and assumed effects on the PVs are left unspecified The emphasis is on System Description and ecosystem aspects Biodiversity, Relations and Ecological Processes and Anthropogenic Processes

^a^The EBM process in BA used material from the previous MAB document, which we therefore included in our analysis

#### Systems thinking

Systems thinking, here meaning inclusion of all ecosystem aspects and recognition of interactions between these aspects, was evaluated as ‘medium’ to ‘high’ score in the analyzed plans. Generally the most specified ecosystem aspects were Biodiversity and Anthropogenic Processes, which can partly be explained by the use of Open standard^5^ procedure for developing the management plans. In the Open standard tool there is a focus on identifying preservation values and impact factors which ultimately translate to Biodiversity and Anthropogenic Processes in this assessment framework. In addition, these are also traditional counterparts in the discourse of nature conservation, presenting nature as a set of values and human activities having mostly negative impact on these values. In the plans of HK, SNS, NB (first column in SM-S3a, b, e), the critical relations and interdependencies between biodiversity and human activities were not specified and hence the integrated view and identification of conflicting goals within the system, which is the core of EBM, was not achieved.

Overall the degree of specificity in the Systems Description management phase was much lower regarding the ecosystem aspects that require a more in-depth systems thinking; Relations and Ecological Processes, Changes and Uncertainty, and Scales (SM-S5). In some of the plans certain relations and processes were acknowledged, but in no plan were this ecosystem aspect comprehensively described. Often, change as depicted in the System Description was historical and/or current trends while future changes or the challenges of managing an ecosystem under constant change were only addressed with a high specificity in the plan of NB (SM-S3, S5e). Climate change was mentioned in the introduction of all plans but not further evaluated. Lack of knowledge of the system was addressed in all the plans and can be interpreted as a notion of uncertainty. Generally no other aspect of uncertainty was considered in the plans, and the System Descriptions were based on present knowledge without discussion of key uncertainties. Only in the plan of HK (SM-S3a) was the need for continuous monitoring and revision of management highlighted in the Systems Description.

Scales and especially cross-scale interactions are at the core of systems thinking since it relates to all the other ecosystem aspects and their inter-linkages (SM-S1b). In the case study this was clearly the most difficult ecosystem aspect to address. There was a marked dominance of the species level of biodiversity, genetic diversity was mentioned only in the plan of BA, while the biotope level of biodiversity was recognised to a varying degree in the plans. The more advanced systems thinking included descriptions of the interaction between species and biotopes in for example food webs and dispersal patterns. Several of the plans used maps to illustrate the spatial distribution of some ecosystem aspect (populations or vegetation coverage), but in none of the plans these maps were overlaid in order to identify areas of specific importance, severe impact or conflicting interests. Temporal scales were addressed to a very limited degree and mostly as description of system history. There were no formulations regarding fast and slow changes in the systems.

#### Specificity and integration

Overall the highest specificity concerned the management phase System Description and the ecosystem aspects Biodiversity and Anthropogenic Processes (SM-S5), which were given the most weight in the plans. All the plans scored ‘medium’ regarding specificity except for SNS (SM-S3b, S5b) that scored ‘low’ because of the strong focus on biodiversity preservation with limited recognition of other ecosystem aspects throughout the plan. HK and SAM showed ‘medium’ specificity for almost all ecosystem aspects and across management phases (SM-S3a, c, S5a, c) whereas BA and NB displayed a larger variety and higher specificity regarding Anthropogenic Processes, ‘medium’ regarding Biodiversity and Changes and Uncertainty and ‘low’ regarding Relations and Ecological Processes, and Scales (SM-S3d, e, S5d, e).

The average degree of integration across the ecosystem aspects was ‘medium’ to ‘low’ and no plan showed a high degree of integration (SM-S5). The patterns of disintegration across management phases varied greatly. In the SNS plan the ‘high’ degree of specificity and content in the System Description was not matched in the other management phases (SM-S3b, S5b). In SAM the main gap in the management was the lack of a monitoring program, as well as the lack of recognition of scales and very general formulations of goals that did not match the specificity in other management phases (SM-S3c, S5c). In BA the content and degree of specificity of the System Description and in Monitoring/Evaluation phases was high, but these were not matched in the Goals phase or the suggestions in Strategies/Measures phase, resulting in a ‘low’ degree of integration throughout the plan (SM-S3d, S5d). In HK and NB the integration scored ‘medium’ and can be interpreted as on its way however with certain gaps and mismatches across the management phases (SM-S5a, e).

#### Assessing the EBM progress

From Table 3 some points of discussion regarding EBM progress can be highlighted.

SAM and HK seem to be on the way toward EBM having a ‘medium’ score for system thinking, specificity and degree of integration. The average scores are in these cases not helpful for further improvement, it is instead necessary to use the higher-resolution analysis matrices (SM-S3, S5) to identify relevant points of intervention for further progress toward EBM. The challenge for the NB plan is to translate the well-developed system thinking into an operational management by increasing overall specificity in the other management phases as well as integration between those.

BA is a specific, but not unique, case where the EBM process was conducted in parallel to the Man and Biosphere Area (MAB) designation that required a very detailed system analysis. The management plan is related to the MAB, but there is a large difference in degree of specificity between the System Description and Monitoring/Evaluation phases in the MAB documents in comparison to the Goals and Strategies/Measures phases as depicted in the management plan, and hence the ‘low’ score for integration. Two of the other cases, HK and SNS are assigned to other formal institutions (UNESCO World Heritage and RAMSAR), but these processes are not used in the management plans. For progress toward EBM in BA it is desirable to further integrate the two processes and especially make better use of the MAB material across management phases.

SNS was the area of the five that showed least progress toward EBM, due to ‘low’ specificity and integration across management phases. Like SAM, it is one of the smaller areas in the study and with few permanent residents. SNS is located at distance from more intensive human activities and the main human activity is outdoor recreation. In the plan the preservation goals are prioritized over human activities, which are described as small scale outdoor recreation activities, and the impact factors identified are described as external and beyond the reach of the management plan. It can be questioned if EBM at this scale is a relevant framework for managing the area. There might also be other explanations to the ‘low’ degree of specificity and integration related to the process of plan development (Sandström et al. 2014).

A well-developed and specified presentation of the management phase Monitoring/Evaluation is a reflection of how well the system thinking is operationalised. Evaluation is essential to enable adjustments of management to changes in the system. There was a great variation in monitoring systems among the five plans. While the HK and BA plans included well-developed monitoring systems (a ‘high’ degree of specificity), one suggested measure in the SAM plan was to develop a monitoring system (SM-S3a, c, d).

## Discussion

Our assessments of the five EBM plans highlight some general points that exemplify the use of the assessment framework. Our analysis suggests that achieving high scores for specificity and integration is the most difficult part of EBM. Without underestimating the challenge of reaching good understanding of the system to be managed, it seems even more challenging to turn those insights into coherent, integrated and well specified goals, strategies, measures, monitoring and evaluation activities. This indicates that the ability to deliver good results on this core feature of EBM is still underdeveloped despite several decades of scientific and policy-practice elaborations about EBM.

Another reflection from our results is that goals and measures are often defined on a scale which is not well aligned with the scale of the EBM target area (cf. Folke et al. 2007). Four plans highlighted the large scale eutrophication that has been identified as a key environmental problem in the Baltic Sea, resulting from, among other things, nutrient rich run-off from agricultural lands and insufficient sewage treatment. However, one of the areas, SNS, represents a group of small and remote islands where these activities are nearly non-existent (SM-S2), yet eutrophication is a major issue with impact on identified values. In this case it might be questioned whether the management of this small geographical area, as delineated in the previous to the planning process, is appropriate for EBM since it is too small in comparison to one of the dominating ecological processes. The delimitation of the target area is of key importance to any EBM process. The boundary needs to be kept flexible so it can be adapted to new knowledge on ecological processes and/or match changes in the overall social–ecological system.

Our aim was to provide a high-resolution assessment framework focusing on the ecosystem aspects of EBM, and in particular how well these are transferred into management practices. Its focus is set on if, and to what extent, an ecosystem perspective is applied and turned in concrete and measureable management activities. Although the framework is highly interdisciplinary, it is not intended as a framework for a complete assessment of all criteria defining EBM as of today (e.g., the Malawi principles, SM-S1). Given the conceptual differences and the high number of criteria currently used to define EBM, we believe that it would neither be feasible nor desirable to develop an analytical framework that covers all aspects of EBM. Therefore our tool must be seen as one in a toolbox to be used when evaluating EBM. Even though our framework is intentionally limited in scope in comparison with the breadth of the EBM concept, it can accommodate significant contextual variability, yet still deliver comparable results. In the specific cases evaluated here, most of the data came from the management plans produced by each of the five regional processes. However, the framework itself does not prescribe certain types of input data, nor does it prevent mixing different types of data sources. The analytical framework specifies what ecological and social/managerial aspects to look into (Fig. 1) and, in this paper, we have provided general criteria for how to assess these aspects (Table 1). The empirical investigation can, however, be done by other means than document studies; using interviews, surveys, observation, etc. It is also possible to break out, and examine, only some parts of the assessment matrix (Fig. 1).

An advantage of the framework is that it can be used to evaluate both single and multiple cases of EBM. When evaluating a single case, it is particularly important to interpret and define the assessment criteria and their different scores in a well-informed and context relevant way, making sure that the definitions of what constitutes ‘low’, ‘medium’ and ‘high’ (Table 1) are discussed together with the assessment results. A cross-case comparative analysis focusing merely on the differences between the cases is however less reliant upon a detailed discussion on why a certain degree of, for example, integration should be denoted ‘high’ or ‘medium’, but nonetheless it is important to define criteria so as to allow meaningful comparisons among cases.

Our framework is inherently high-resolution since the studied object is evaluated on an ecosystem aspect-by-management phase approach. The assessment matrix (Fig. 1) has many cells that need to be filled with content before any higher-level order of analysis can be carried out. Similarly as when contextualizing the details of the assessment criteria, a higher-level order analysis such as integration also benefits from an iterative approach going back and forth between evaluating fine grain details and systemic properties. As a consequence of this assessment process one can freely choose the level of aggregation for further evaluation of the results. For example, in Table 3, we compare the five cases on an aggregated level, i.e., we only compare their unitized scores of the overall criteria of systems thinking, specificity and integration. However, as a result of the assessment process, we could have chosen to compare the cases on a more fine-grained level, down to cell-by-cell comparison using the assessment matrices (SM-S3). Thus, the framework allows for analysis on different levels of resolution, enabling both detailed and aggregated analyses.

In summary, we argue that the analytical framework is useful by providing a meaningful, transparent, and fairly robust assessment process for the multi-facet concept EBM. It has plenty of room for further refinements, and would naturally benefit from being applied in more cases by different researchers. For example, during the analysis we found it challenging to keep the assessment criteria specificity and integration separate. A well-motivated assessment of the level of specificity for each cell in the evaluation matrix depends, to some extent, on the level of integration for ecosystem aspect. In other words, a certain level of specificity, for a specific management phase and ecosystem aspect pair, makes most sense if it is set in accordance with the level of specificity for the other management phases of that ecosystem aspect. Thus, in the end, the assessments of specificity and integration are not completely independent. Although this is a limitation of the framework, we nonetheless argue that these criteria should be kept apart to the extent possible. The reason is that it is theoretically important to distinguish these features since they may be the effect of, for example, different aspects of the collaborative (and/or conflicting) processes actors engage in when charged with the task to jointly develop EBM.

Even acknowledging these limitations, the framework provides a basis for a refined analysis of how to improve EBM in any given case. The literature on why it is good and desirable to aim for EBM abounds and is highly convincing, but it seems like the EBM concept itself expands at a much higher pace than knowledge of how to accomplish EBM accumulates. Research on successful transformation toward EBM requires assessment criteria that take into account that EBM is a multi-facetted and constantly developing framework, and allows success to be assessed individually, using different types of empirical data. We believe the assessment framework to be of particular use for longitudinal studies of governance transformations that continually assess the progress in adopting EBM, while also simultaneously keeping track of possible explanatory factors and hence key points for improvements. In all, this also implies that the assessment framework could provide valuable inputs for policy-makers and practitioners when evaluating the processes of different EBM programs and initiatives.

## Electronic supplementary material

Supplementary material 1 (PDF 944 kb)

## Footnotes

1 The SEPA has also evaluated the plans produced by these five areas, but used different assessment criteria and the procedures. Thus, the results from these two evaluations are not fully comparable (SEPA 2011).

2 For more information see: https://miradi.org/openstandards.

3 Atlas.ti is a software for qualitative data analysis using texts, recordings, pictures etc. More information is found on: www.atlasti.com.

4 For more information see: https://miradi.org/openstandards.

5 For more information see: https://miradi.org/openstandards.
