# Supplementary material for: Developing an analytical framework for assessing progress toward ecosystem-based management
Source: Ambio. 2015 May 28;44(Suppl 3):357–69. doi: 10.1007/s13280-015-0655-7 (PMC4447704; doi:10.1007/s13280-015-0655-7)
Supplement: Supplementary file 1 — Supplementary material 1 (PDF 944 kb) [file 13280_2015_655_MOESM1_ESM.pdf]

AMBIO

Electronic Supplementary Material

This supplementary material has not been copy edited by the publisher or the editorial office

Title: **Developing an analytical framework for assessing progress towards ecosystem-based management**

Authors: Sara Borgström, Örjan Bodin, Annica Sandström, Beatrice Crona

Supplementary Material S1a

The Malawi principles (ecological principles in *Italic*)

1) Management objectives are a matter of societal choice.
2) Management should be decentralized to the lowest appropriate level.
3) *Ecosystem managers should consider the effects of their activities on adjacent and other ecosystems.*
4) Recognizing potential gains from management there is a need to understand the ecosystem in an economic context, considering e.g. mitigating market distortions, aligning incentives to promote sustainable use, and internalizing costs and benefits.
5) *A key feature of the ecosystem approach includes conservation of ecosystem structure and functioning.*
6) *Ecosystems must be managed within the limits to their functioning.*
7) *The ecosystem approach should be undertaken at the appropriate scale.*
8) *Recognizing the varying temporal scales and lag effects which characterize ecosystem processes, objectives for ecosystem management should be set for the long term.*
9) *Management must recognize that change is inevitable.*
10) *The ecosystem approach should seek the appropriate balance between conservation and use of biodiversity.*
11) The ecosystem approach should consider all forms of relevant information, including scientific and indigenous and local knowledge, innovations and practices.
12) The ecosystem approach should involve all relevant sectors of society and scientific disciplines.

Supplementary Material S1b

Scientific rational and detailed content behind each of the five ecosystem aspects (Fig 1)

An ecosystem is defined as interacting biotic and abiotic compartments that also interact with the surrounding (Tansley 1935) and this is occurring at several and often interlinked spatial and temporal scales. The biotic compartments are today usually described in terms of biological diversity at different levels of organisation, genes, species and biotopes/landscapes. Within in EBM this means recognising different levels of biodiversity, such as quantities, proportions and scarcities, and also the dynamics such as population variation, structural composition and succession stages (Malawi principle 5). The next system characteristic to address in EBM is the interaction between the compartments, here termed relations, such as food webs dynamics between species or species groups, and interaction between biotic and abiotic compartments, e.g. population dynamics that could be related to hydrological variations or topography and humidity impacting species competition, species community viability and distribution (Malawi principle 5). Crucial to a system functioning is also ecological processes such as disturbance regimes, e.g. forest fires, and biogeochemical cycles of for example water, nitrogen and carbon. Current research describes ecosystems as complex adaptive systems and highlights the importance of disturbances and change for ecosystem resilience (Christensen et al. 1996; Levin 1998; Folke 2006; Gunderson and Holling 2002) and hence a need for any management scheme to handle a high degree of uncertainty, such as cascading effects or regime shifts, and allow for and adapt to these abrupt or gradual reoccurring changes (Malawi principle 9, Christensen et al.1996; Walker et al. 2004). Furthermore the temporal and spatial scales of operation and dynamic of the ecosystem compartments, relations and processes, are essential to understand the systems functioning (Malawi principles 3, 7-8, Christensen et al., 1996; Peterson et al. 1998; Gunderson and Holling 2002). Examples are population dynamics within and between species over time, species community successions, disturbance regime cycles and spatial distribution of biotopes and processes. Another aspect of scales is the process of area delimitation within any management, which refers to the sixth Malawi principle of EBM requiring that the management matches the spatial scales of the ecosystem and the processes that makes it functional (Cumming et al. 2006). Hence to succeed in all other aspects of EBM this is a crucial step. Lastly, no ecosystem

can be analysed without counting for human activities interact with ecological structures and processes (Christensen et al. 1996; Berkes and Folke 1998) , ranging from negative impact (environmental degradation) to necessary human involvement in ecological processes for sustaining ecological functions (e.g. wetland restoration schemes, management of biodiversity rich semi-natural grasslands and protection of areas). Thus recognition of anthropogenic processes is an essential part of EBM (Malawi principles 6, 10). Crucial to EBM designs is also the balancing act between interests of use and preservation of natural resources and ecosystems which is a matter of navigating social, economic and ecological processes that all are part of the systems' dynamic, e.g. fisheries, forestry, biodiversity conservation and tourism. To fulfil an EBM all these aspects must be addressed.

Supplementary Material S2
Description of case study areas.
The five areas included in the Swedish EPA initiative of integrated coastal and marine management planning. See also the area location in figure 2, referred to as "A, B, C, D, E" in the table. (For further details see Sandström et al., 2014)

	**Höga kusten, HK,"A"**	**Stora Nassa-Svenska högarna, SNS, "B"**	**St Anna-Missjö, SAM, "C"**	**Blekinge Arkipelag, BA, "D"**	**Norra Bohuslän, NB, "E"**
**Total Area (ha)**	152 034	29 852	16 610	210 000	539 700
**Share ocean**	53 %	98 %	94 %	74 %	60 %
**Share formally protected**	5 %	19 %	55 %	10 %	29 %
**Population (permanent = p, part time, pt)**	5400 (p), 2400 (pt)	2 families	4 (p)	85 000 (p)	58 000 (p) 40 500 (pt)
**International status**	BSPA + UNESCO World Heritage area	BSPA + RAMSAR	BSPA	BSPA + UNESCO MAB area	MPA
**No of municipalities**	2	2	1	3	5
**Identified critical issues**	Aquaculture, shoreline exploitation, invasive species	Boating, Fish re-generation, large scale environmental impacts	Preconditions for permanent population, fishing, tourism and outdoor recreation	Sustainable business development, preconditions for island populations, fishing, tourism and outdoor recreation	Sustainable development and tourism, fishing, boating and aquaculture
**Vision**	A healthy ocean is interlinked with viable populations of plants and animals and ecosystems in balance. A living archipelago means a living environment for humans with housing, businesses, service, tourism and infrastructure.	Preservation of the unique character of the environment in parallel with sustainable use of the area.	A genuine outer archipelago environment , shaped by physical preconditions and centuries of archipelago culture and living	A living coast and archipelago where the development is in harmony with entrepreneurship and ecology. The base is the local engagement and care for future generations.	A balance between different interests for a sustainable use of a unique coastal and archipelagic environment.

Supplementary Material S3
Assessment matrices for management plans

Abbreviations: ad: addresses, D: description, G: Goals, S: strategies and measures, M: monitoring and evaluation. IF: impact factors, PV: preservation values, in **bold –** generalised information.

**A) HÖGA KUSTEN (HK)**		**MANAGEMENT PHASES**			
		**System Description**	**Goals**	**Strategies/ Measures**	**Monitoring/ Evaluation**
**ECOSYSTEM ASPECTS**	**Biodiversity (Genetic, Species, Biotopes)**	-5 PVs concern biotopes (4 water environments) and 1 PV concerns sea birds<br>- 5 fish species (ecology and status) and 5 bird categories<br>-IFs: invasive genes and species, bird disturbance and migration barriers, unsustainable fishing and fish diseases<br>- beaver, mink, insects, newts, crayfish, water plants, N2000 species<br>- bladder wrack as key species<br>- sub-biotopes are mentioned, e.g. lagoons<br>- terrestrial biotopes<br>- cormorant and seal problematic is not ad since it concerns a "social" value	- delimited existence and introduction of invasive genes and species<br>- increased recognition of sensitive bird sites and times<br>- decreased number of migration barriers<br>- increase and conservation of valuable shoreline biotopes	- decrease dispersal of invasive genes and species (hunting of mink)<br>- create protected areas for seabirds<br>- Shorelines: sustainable management and creation of zones	- species indicators for biotopes generally<br>- bladder wrack as key species (indicator/structural)<br>- indicator species fresh water: fresh water clam, 2 fish species, otter<br>- indicator species in marine biotopes: macro-algee distribution sea floor fauna<br>- seabirds indicators: 2 predatory birds, coastline birds, shoreline meadow birds<br>- shoreline indicators: increase of area, pop of beetle and bird
	**Relations and Ecological Processes**	-relations: links btw biotopes for species viability, the importance of bladder wrack for the systems functioning, the importance of shallow bays for the sea ecosystem at large, species relation to abiotic factors<br>-processes: land rise, sea salt level, depth and wave exposure are key to the diversity (change), material transportation and nutrient leakage as IF, migration	- less nutrient leakage (ref to EU Water Framework Directive)<br>- decrease number of migration barriers	- create free migration routes<br>- increase degree of cleaning in waste water plants/private sewers	- indicators are explained in relation to system compartments and interactions, e.g:<br>- changes in vegetation → water quality changes<br>- oxygen level at lake bottom, turbidity, pH value, fresh water mussel, 2 fish species, otter → fresh water biotopes<br>- level of sight → deep marine biotopes<br>- macro-algae distribution and sea floor fauna → shallow/deep marine biotopes<br>- pop of beetle and bird → shorelines
	**Changes and Uncertainty**	- adaptation is ad in intro and need for regular updates of the whole plan<br>- the process of land rise is key to the diversity (change)<br>- knowledge gaps are ad as needs for inventories of IF, abiotic factors, species, environmental quality (uncertainty)	-recognise need for changes in the plan due to new knowledge<br>- changes in priorities due to changed conditions e.g. boating	-recognise need for changes in the plan due to new knowledge<br>- knowledge gaps are ad as needs for inventories of IF, species, environmental quality<br>- all G are followed by measures concerning knowledge generation and planning	-recognise need for changes in the plan due to new knowledge<br>- monitoring aims to estimate changes from a baseline value<br>- each indicator is ad by knowledge status<br>- changes in vegetation indicates water quality changes
	**Scales**	- borders were pre-set and there is a World Heritage Area with same	- increased recogn. of sensitive bird sites and times	- create protective zones around shorelines	-not ad

		borders<br>- is limited to the marine environment excluding terrestrial<br>Spatially explicit (maps) concerning:<br>- abiotic factors<br>- fish habitats for reproduction<br>- species distribution<br>- some anthropogenic processes<br>- invasive species and genes	- not ad temporal scales	- not ad temporal scales	
	**Anthropogenic Processes**	- ad as impacts on the marine environment<br>- ad as important for the area: outdoor recreation, businesses, commercial fishing, aquaculture<br>- high priority IF: shoreline exploitation, material transportation, nutrient leakage<br>- low priority IF: acidification, dredging, transport of hazardous material, oil pollution, unsustainable fishing, insufficient management of semi natural grasslands, change of water flows, spread of fish diseases, waste disposal	- less nutrient leakage, increase degree of cleaning in waste water plants/private sewers<br>- decrease of toxic pollution<br>- decrease impact from outdoor recreation on seabirds<br>- increase awareness of areas sensitive to dredging<br>- less impact from sea based aquaculture<br>- sustainable use of shoreline environments, develop sustainable boating	- less nutrient leakage, increase degree of cleaning in waste water plants/private sewers<br>- decrease of toxic pollution<br>- decrease impact from outdoor recreation on seabirds<br>- increase awareness of areas sensitive to dredging<br>- less impact from sea based aquaculture<br>- sustainable use of shoreline environments, develop sustainable boating	- area of nature valued areas → degree of human impact<br>- level of sight indicator of deep marine biotopes<br>- oxygen level at lake bottom, turbidity, pH value as indicators in fresh water biotopes (Ref. to Water Framework Directive)

**B) STORA NASSA – SVENSKA HÖGARNA (SNS)**		**MANAGEMENT PHASES**			
		**System Description**	**Goals**	**Strategies/ Measures**	**Monitoring/ Evaluation**
ECOSYSTEM ASPECTS	**Biodiversity (Genetic, Species, Biotopes)**	- genetic div is not ad<br>-species: threatened plants, vegetation cover species, newts, vipers and toads, birds, fish, seals, algae<br>- different islands and their resp. surroundings, e.g. many different biotopes and species<br>-soft and hard bottoms and shallow bays are ad as important for species regeneration	- genetic div is not ad<br>- generally ads in overarching goals (e.g. marine habitats, deep and shallow sea floor habitats in PVs)<br>- 1 fish species and 5 bird species ad in relation to PVs<br>-no terrestrial species are targeted and terrestrial environments are not ad as consisting of different biotopes	- genetic div is not ad<br>Restoration of predatory fish population (pike)<br>Inventories of fish populations<br>Inventories of bird species, migration patterns<br>Mink hunting<br>Inventories of bottom habitats<br>Shoreline measures in terms of restrictions for exploitation	- genetic div is not ad<br>-bladder wrack as an indicator species<br>-evaluation of pike reintroduction measures<br>-inventories of bird species, migration patterns<br>-observation of flora/fauna both marine and terrestrial, mink<br>-continued monitoring/Inventories of seafloor habitats and water quality
	**Relations and Ecological Processes**	-relations btw biotope compartments, btw geomorphology, biotopes and species occurrence<br>-the importance of shallow bays for fish pop regeneration<br>-dynamics btw bird and fish population	- goals addressing relations btw fish and bird populations<br>-Ecological processes are not ad	-food webs are ad (restoration of predatory fish population and mink hunting)	x

		(food webs)<br>-processes: wave exposure, succession of vegetation cover across a gradient of islands, nutrient cycling, over growth of semi natural grasslands			
	**Changes and Uncertainty**	-climate change, population dynamics over time<br>- lack of knowledge esp of population dynamics and causal relations in the system	-goals are discussed as temporary due to uncertainty about system dynamic and change (e.g. the decline of eider)	-generally ad as a need of knowledge generation	-generally ad as a need for continuous revisions of the plan in relation to change and new knowledge
	**Scales**	- borders were pre-set to the planning process<br>- ads that the area is part of and interrelated to a larger system as well as consisting of smaller subsystems<br>- most IF are discussed as external and beyond the reach of the plan<br>- previous land use as well as future trends (e.g. fish and bird species population changes) are ad	-some goals are spatially delimited since the main IF is at a larger spatial scale than the reach of the plan	x	x
	**Anthropogenic Processes**	-Climate change, overfishing and eutrophication is identified as major and external IF<br>-Visitor frequency is highlighted as an locally important IF<br>- The need of management of semi-natural grasslands	-large scale IF are ad as beyond the scope of the plan<br>- sustain management of semi natural grasslands<br>- sustainable tourism and delimit negative impacts from visitors	-continued management of semi-natural grasslands<br>-survey visitor patterns and behaviour to increase knowledge<br>-create protected areas	-Monitor trolling impact on marine species and biotopes<br>-Survey visitor patterns and behaviour

C) ST. ANNA-MISSJÖ (SAM)		MANAGEMENT PHASES			
		System Description	Goals	Strategies/Measures	Monitoring/ Evaluation
ECOSYSTEM ASPECTS	**Biodiversity (Genetic, Species, Biotopes)**	**- genetic diversity is not ad**<br>**- species/biotopes ad in detail and quantitatively**:<br>- 2 PV at species level, 5 at biotope level<br>- seals and birds in detail and over time<br>- seafloor fauna + vegetation<br>- lists of threatened species<br>- number of plants, insects and birds in different terrestrial biotopes<br>- species communities in forest sub-biotopes<br>- population status of	**- genetic diversity is not ad**<br>**- quantitative for species, relative for biotopes**<br>- 2 PV at species level, 6 at biotope level<br>- number of seals, otters<br>- pop of 5 coastal birds<br>- no increase in 2 invasive species<br>- spatial distr. of 2 indicator species And 1 community of sp.<br>-viable pop of forest species (threatened and sensitive)<br>- 5 fish species in good status, 3 species	**- genetic diversity is not ad**<br>**- main strategies for species and biotopes are protection of areas and change of management schemes**<br>- predator control at bird islands<br>- hunting mink, cormorants and seals<br>- protection of species and habitats by creating protected areas<br>- temporal protection of fish reproduction areas<br>- create biotopes for nutrient retention<br>- regulation of forestry and sustainable	**- genetic diversity is not ad**<br>**- quantitative indicators are suggested for species, communities and biotopes**<br>- number and health of seals and otters<br>- colonies on bird islands and number of breeding pairs of threatened birds<br>- indicator species for biotopes<br>- shallow marine species communities as indicator system for water quality<br>- fish species age structure<br>- number of cormorant

		fish species in relation to threats<br>- invasive species as IF<br>- marine biotopes are described in very quantitative terms	reproduction goal<br>- less damage from seals<br>- mink population is no threat<br>- grasslands in good management and increasing<br>- sustained area grazed forest<br>- small scale forestry with nature conservation perspective.	management agreements<br>- enhanced management of semi natural grasslands	colonies<br>- degree of seal damage<br>- no monitoring of invasive species<br>- forest age structure and area of certain forest types<br>- area managed semi natural grassland
	**Relations and Ecological Processes**	**- relations ad; food webs and species-abiotic relations**<br>**- processes ad; tree succession, species migration, seed dispersal, nutrient cycling**<br>- toxic substances and eutrophication impacts food supply for species<br>- linkages between birds and flora on bird islands<br>- IFs relating to processes: eutrophication, over growth, species migration, tree decomposition, grazing regimes, seed dispersal, landscape fragmentation<br>- fishing is not mentioned as impacting food webs	**- some species relations are ad**<br>**- quantitative goals for some of the processes (succession, decomposition, nutrient cycling) and relations btw species**<br>- good water quality and good chemical water status (ref. to the EU Water Framework Directive)<br>- fish migration routes are accessible<br>- grasslands in good management and increasing area<br>- high share of old trees and dead wood	**- general biotop preservation**<br>**- measures that target food webs, migration and nutrient retention**<br>- create wetlands and mussel banks<br>- information/regulation for nutrient retention<br>- hunting of mink<br>- remove fish migration barriers<br>- protection of habitats in protected areas<br>- regulation of forestry and sustainable management agreements<br>- management of semi natural grasslands	**- quantitative indicators are suggested for some of the processes (succession, decomposition, nutrient cycling) and for one species relation**<br>- water quality (ref. to the EU Water Framework Directive)<br>- degree of sedimentation<br>- degree of over growth of semi natural grasslands<br>- amount of dead wood<br>- quota between fish species
	**Changes and Uncertainty**	**- previous and future changes are ad as part of system development (species, resource use, landscape, IFs)**<br>**- climate change is mentioned but not ad**<br>**- an adaptive approach as well as evaluation schemes are suggested but in general terms**<br>**- uncertainty is ad in terms of knowledge gaps concerning both PVs and IFs**	**- general goals of increase knowledge**<br>**- not ad need of revision of goals**	**- does not ad how to become more adaptive**<br>**- many measures concern knowledge generation (mapping, inventories and evaluation)**	**-change is implicitly ad. by suggesting indicators, that can be followed over time, however not explicitly ad.**<br>**- no monitoring of knowledge increase and its potential effects**
	**Scales**	**- borders were pre-set to the planning process**<br>**- the area is ad as part and dependent on a larger system**<br>**-different spatial scales are ad, not cross scale interactions**<br>**-historical land/water use, population trends, future prognosis**	**- spatial and temporal scales are not ad, except for fish reproduction sites**	**-knowledge generation for optimising spatial division of fishing and fish conservation**<br>**- time priorities are administrative, not ecologically based**	**- no indications of where or when monitoring is going to take place, and not related to ecological scales**

		- spatially defined biotopes<br>- marine PVs are spatially and temporally explicit<br>- trends over time for some PVs and IF, not all<br>- maps of distribution of biotopes, habitats and exploitation			
	**Anthropogenic Processes**	**-ad as natural resource management or use and as IF,**<br>**- seen as necessary for the sustained PVs,**<br>**- in detail, often quantitative**<br>- fishing, agriculture and forestry, tourism, boating and outdoor recreation as IFs and of importance for PVs<br>- eutrophication (external)<br>- invasive species (external)<br>- oil spill (external)	**- very general and some prohibitions**<br>-Sustainable boating<br>-Sustainable fishing<br>-Sustainable tourism<br>-No clear-cutting<br>-No dredging or dumping of material<br>-Low degree of exploitation	**- very rigorous and detailed and most strategies concern these processes**<br>- regulation of forestry and sustainable management agreements<br>- regulation/organisation/ knowledge generation of fishing<br>- PVs integration into physical planning<br>- planning, information, education, co-operation, advice, regulation for more permanent inhabitants and sustaining economic activities → sustain PVs	**-very few indicators, rather suggesting indicators that measure the effect of reduced anthropogenic processes**<br>- number of landings, non-built islands, extensions of fire damages and wear from visitors<br>- number of animal keepers, stables and cattle

**D) BLEKINGE ARKIPELAG (BA)**		**MANAGEMENT PHASES**			
		**System Description**	**Goals**	**Strategies/ Measures**	**Monitoring/ Evaluation***
**ECOSYSTEM ASPECTS**	**Biodiversity (Genetic, Species, Biotopes)**	**- genetic diversity is ad in D (cattle, crops)**<br>**- all PVs described as biotopes (excl. fish) and are related to species**<br>-threatened species (insects, fungi), species of economic importance (fish) and species of structural importance (trees, macroalgees)<br>-Invasive species as IF<br>- 9 biotopes are described in detail in the MAB application	**- genetic diversity is not ad**<br>**-relative, some quantitative,**<br>**-diff in level between marine and terrestrial: marine species, terrestrial biotopes**<br>- enhanced seafloor flora and fauna and status for phytoplankton<br>- increase in fish and bird populations<br>-sustained population of seals, toads (redlisted), insect (redlisted)<br>-sustained habitats for amphibians and natural wetlands and increased establishment of wetlands<br>-sustained large trees<br>-restore and increase the area semi natural grasslands<br>-increased area broadleaved forest and sustainable forestry	**- genetic diversity is not ad**<br>**-rather general, not explicitly addressing the details in D and G**<br>- fish: actions for sustainable fishing, restoring and protecting reproduction sites, removal of migration barriers<br>- Restoration and continued management of semi natural grasslands<br>- Actions for enhanced forest biodiversity<br>- Establish wetlands for biodiversity and nutrient retention<br>-Sustain and restore meandering streams	**- genetic diversity is not ad**<br>**- is not matched to D, G, S, esp terrestrial biotopes and species are missing in this function**<br>-marine vegetation and frys in shallow bay environments<br>-otter and newts population<br>- algae communities<br>- shoreline birds and birds in forest/agricultural landscape<br>- effects of new wetlands<br>- key forest biotopes
	**Relations and Ecological Processes**	**-processes and relations are rigorously and detailed, linked to each biotopes**<br>-food webs (fish/seals)<br>-fish populations and microalgae as important for marine ecosystem	**-very general comp to D**<br>-Improved nutrient status in water<br>-Less regional eutrophication (ref. to EU Water Framework Directive)<br>-Restore and increase the area semi natural grassland	**-just a few processes are ad and no relations**<br>-Actions for continued management of semi-natural grasslands<br>-Actions for minimizing nutrient	**- nearly absent except for water quality (chemical status and effect of new wetlands)**<br>(ref. to EU Water Framework Directive)

		functioning<br>-wetlands important for nutrient retention<br>-over-growth of former semi natural grasslands<br>-species migration<br>-nutrient circulation and hydrological cycles<br>-linkages between biotopes are not ad<br>- gap dynamics and disturbance regimes in forests<br>- water level variations, decomposition, erosion, connectivity		leakage<br>-Removal of migration barriers	
	**Changes and Uncertainty**	**- overall adaptive approach is ad in introduction<br>- change is ad as part of the biotope dynamics<br>- climate change is ad as an IF but it was not included in the process**	**- ad as a need of continuous update**	**- ad as a need of continuous update<br>- many measures concern knowledge generation to decrease uncertainty**	**-no monitoring explicitly targeting change or increase of knowledge per se**
	**Scales**	**- borders and the plan are related to the application for UNESCO MAB area<br>-spatial and temporal scales are ad (historical land/water use, population trends, future prognosis)**	**-spatial scales implicit ad as areal coverage and distribution<br>- temporal scales are not ad**	**-spatial scales implicit ad as areal coverage and distribution<br>- temporal scales are not ad**	**-spatial scales implicit ad as areal coverage and distribution<br>- temporal scales are not ad**
	**Anthropogenic Processes**	**-ad as part of the system dynamic and in relation to each biotope, necessary for the PVs sustainability, detailed**<br>- unsustainable fishing<br>- eutrophication<br>- pollution (air and water)<br>- exploitation, dredging, drainage<br>- noise<br>- migration barriers<br>- tress cutting<br>- wear from visitors<br>- climate change	**-rigorous and detailed concerning social factors of importance to decrease negative impact**<br>-less regional eutrophication<br>-sustainable exploitation, forestry, agriculture, boating, fishing<br>-less pollution<br>-sustainable fisheries<br>-increase of inhabitants	**-rigorous and detailed concerning social factors of importance to decrease negative impact**<br>-actions for continued management of semi natural grasslands<br>-actions for integrated planning of development<br>-actions for minimizing nutrient leakage from private and public sources, from forestry and agriculture<br>-actions for sustainable tourism, boating and fishing	**-not explicitly ad. (exp. monitoring of attitudes)<br>- implicit in water chemical status**

** This phase is to its full extent found in an external material concerning the MAB area management. The EBM plan and the MAB plan are explicitly linked.*

**E) NORRA BOHUSLÄN (NB)**		**MANAGEMENT PHASES**			
		**System Description**	**Goals**	**Strategies/ Measures**	**Monitoring/ Evaluation**
**ECOSYSTEM ASPECTS**	**Biodiversity (Genetic, Species, Biotopes)**	-genetic level is not ad<br>-2 PVs ad mammals/birds, fish/shellfish<br>-3 PVs ad shorelines, shallow and deep marine systems<br>- seals, porpoises, birds, fishes<br>- threatened species in shallow marine system<br>- macro algae as sub-	-genetic level is not ad<br>-population goals of cod, seal, bird<br>- limited impact from invasive species, mink<br>-distribution goals of species communities at seafloors<br>- less overfishing (by changing gears and methods) and damage on	-genetic level is not ad<br>-actions for sustainable fishing (less bi-catches, spoke fishing, enhanced selective fishing)<br>- actions for sustainable aquaculture<br>- mink hunting	-genetic level is not ad<br>- monitoring of sea floor fauna, phytoplankton, macro algae<br>-inventories of birds and seals<br>-hydrography

		biotope<br>-vascular plants in shoreline biotopes<br>-plant as an invasive species (IF)	seafloors<br>- less damage on seafloors (from construction, boating, tourism)<br>-no shoreline exploitation or oil pollution<br>-decrease area anoxic seafloors		
	**Relations and Ecological Processes**	-relations: btw species and biotopes, shallow marine systems as essential for the larger system function, btw shallow and deep biotopes for species viability<br>-processes: nutrient cycling, oceanographic dynamic, hydrology, erosion, succession	-less eutrophication<br>-increased water quality (ref. EU Water Framework Directive)<br>-decreased area anoxic seafloor<br>-increased management of semi-natural grasslands	-reducing nutrient leakage from agriculture, private drains, industries, treatment plants, forestry and boats<br>-enhance nutrient retention and erosion control<br>- sustainable aquaculture practices (mussels for water cleaning)	-water quality (toxins, nutrients, benthic quality index), (ref. EU Water Framework Directive)<br>-phytoplankton<br>-macro algae distribution
	**Changes and Uncertainty**	-climate change effects has been observed → large uncertainties but also expected large scale changes<br>-ad as previous/present changes in species populations	-adaptation to climate change<br>- enhance knowledge, research and information part of nearly all goals	-enhance knowledge, research and information part of nearly all strategies	-not addressed
	**Scales**	- the area is delimited to the marine environment + 300 m shoreline, but also ad the drainage basin<br>- borders related to another coastal zone planning program<br>- spatially explicit in multiple maps (e.g. hydrography, land cover, nature types)<br>-areas are divided into subareas (e.g. soft and hard sea floors)<br>-ad the importance of biotopes for the whole system functioning (e.g. shallow marine systems)<br>- effects of nutrient leakage are dependent on local context	-not addressed	-planning to identify sensitive locations<br>-mapping to find out where and when impact/occurrences/sensitivity/optimisation in relation to strategies	-not addressed
	**Anthropogenic Processes**	-shoreline exploitation (high)<br>-fishing (overfishing/trolling), aquaculture<br>-dispersal of toxins, oil and noise pollution, waste disposal<br>-tourism, wear on land<br>-boating, shipping, seafloor damages<br>-eutrophication	- goals on use of selective fishing gear<br>-less eutrophication<br>- increased management of semi-natural grasslands<br>-less negative impact on climate<br>-no exploitation in untouched and sensitive areas (eg. Energy production)<br>-reduced pollution<br>-create clean and attractive coastline	Actions for:<br>-sustainable fishing (less bi-catches, spoke fishing, enhanced selective fishing)<br>-sustainable aquaculture (less environmental impact)<br>-sustainable energy production<br>-reduced pollution (oil, toxins, noise)<br>-sustainable tourism	-not addressed

Supplementary Material S4
Detailed description of the EBM assessment of the SAM area (C)
(see also assessment matrix in Supplementary Material S3 and score table in Supplementary Material S5)

The SAM management plan process followed the procedure suggested by Open standard[1] with formulation of a vision, identification of preservation values (PV), goals and an analysis of impact factors (IF) and drivers of those. Seven PVs were identified: *marine mammals and coastal birds*, *shoreline and rock environments*, *shallow marine environments, deep marine environments, cultural landscape, forest* and *fish*. 19 IF were identified and analysed in relation to the PVs: *collection of fire wood, migration barriers, leakage of phosphorus and nitrogen, constructions, clear felling in forests, insufficient grassland management, oil spill, unsustainable fishing (professional and recreational), unsustainable boating, dredging, mink population, environmental pollutants, climate change, immigration of invasive species, overgrowth in bays with fish reproduction, fire on rocks, dumping of material and fill ins,* and *lack of management of cultural elements*. The overall goals stated in the plan were to sustain the PVs by decreasing IF. The PVs assessed as most at risk were deep and shallow marine environments and fish. In the introduction of the plan it was stated that a precondition of sustained PVs is socio-economic sustainability and hence most suggested measures concerned social aspects such as how to attract permanent residents and viable businesses to the area. No monitoring program was presented in the plan, but an overview of potential methods for monitoring.

Systems thinking (Score Medium)

The System Description of the plan was explicit and detailed on species, biotopes including sub-biotopes and Anthropogenic Processes, whereas other aspects were less developed. This relates to that the SAM process followed the procedure of Open Standard and both PVs and IFs were described in detail and often in quantitative terms. Generally PVs are related to the biological values identified as important for preservation and IFs are the identified Anthropogenic Processes having impact on the PVs and hence are important to address. Genetic diversity was not addressed, whereas description of Biodiversity at species and biotope level was very detailed and

[1] For more information se: https://miradi.org/openstandards

quantitative, e.g. species lists for different biotopes. Two PVs concerned species (mammals, birds and fish), while the other five, addressed biotope level; both marine and terrestrial. The biotopes in the PVs were also described as consisting of sub-biotopes, e.g. different types of forests. Of the identified IF, mink and invasive species were most explicitly species oriented, while unsustainable fishing more indirectly targeted species level. Some ecological relations were addressed, e.g. linkages between species in food webs and the biotopes where characterised as relations between species and abiotic conditions.

Several ecological processes were addressed both as important parts of the PVs, but also as IFs, where human activities impact these processes. One example was nutrient cycling which is an essential part of both terrestrial and marine biotope dynamics that in this context was only addressed as a key IF in terms of eutrophication, not explicitly as for example an important determinate of biomass production and biodiversity. Other processes addressed were; disturbances in terms of grazing of meadows and pastures for sustained floral diversity, species migration in terms of migration barriers for fish, tree succession of importance for forest species diversity and seed dispersal linking coastal birds to terrestrial diversity. Hence there was a rather high level of understanding of system dynamics regarding the interactions between Biodiversity, Relations and Ecological Processes

Anthropogenic Processes were described in detail and often quantitatively in terms of natural resource management (trends in agriculture, forestry and fishing) or use (boating, tourism) and as IF linked to the PVs. However, even if linked, they were described in separate sections in the plan. Some Anthropogenic Processes were described as necessary for sustained PVs, e.g. small scale farming as essential for terrestrial species diversity. The IFs perceived as most threatening to the system were external; leakage of phosphorus/nitrogen and immigration of invasive species, where there is limited capacity for the present management to act.

Changes in the system, both previous and future, were addressed in the System Description both for PVs and IFs, such as species population variation over time and estimations of future changes of tourism and agriculture. Climate change was mentioned

and depicted as an IF, but not specified in relation to the system or in the rest of the plan. Recognition of temporal scales in the system could be found in the reasoning about change for some PVs and IFs, especially for the marine ones (species population trends). However, the temporal scales were not explicit. Concerning spatial scales the borders of the management area were pre-set and hence delimitation of the area was not part of the process. It was explicitly recognised that this management area was part of a larger system (e.g. taking external IFs into account in the impact analysis). In the System Description the biotopes were spatially defined in maps as well as some IFs (e.g. exploitations). However, there was no recognition of cross-scale interaction, nor overlay of different thematic maps. Uncertainty was addressed in very general terms and as knowledge gaps for both PVs and IFs. In summary the SAM System Description is addressing all ecosystem aspects but to a differing degree of detail and the content is sometimes related between ecosystem aspects. The least detail and content match is found for the ecosystem aspects; Change and Uncertainty and Scales. This equals the score medium.

Specificity (Score Medium)

In the SAM plan the specificity was highest concerning ecosystem aspect Biodiversity across almost all management phases and in the description and measures regarding ecosystem aspect Anthropogenic Processes (Tab 2). For most of the combinations of ecosystem aspects and management phases the specificity was medium. Least specificity was seen for spatial and temporal scales in Goals and Monitoring/Evaluation phases, since this information was missing in the plan.

In the System Description biodiversity was described in detail (except for genetic diversity) as PVs and most of the goals were quantitative and fairly matched to the PVs at both species and biotope level. For example: “by 2020 it shall be at least 10 colonies of herring gulls” and “by 2020 all grasslands shall be in good management and this area should increase from the 2010 level”. The goals formulated at species level to a dominating part concerned marine species and less terrestrial. In the System Description the PV fish is explained by the use of species lists and presentations of present population status as well as change over time in quantitative terms. This is also related to spatial distributions of marine sub-biotopes and anthropogenic processes.

The same level of specificity is seen in the quantitative population goals for five fish species and reproduction goals for three and is also seen in the suggested monitoring. But there is a lower degree of specificity in the presentation of measures to fulfil the goals. Predator control and areal protection are suggested measures but these are not presented in quantitative terms and neither spatially nor temporally explicit.

A large part of the plan focused on in detail description of identified IF (Ecosystem aspect Anthropogenic Processes) and the measures aimed to decrease these and thereby sustain identified PVs. Such high degree of specificity was not found in the other management phases regarding Anthropogenic Processes. The description was detailed and quantitative (e.g. number of visitors, incomes from tourism, boating frequency) and was matched by equal level of specificity in suggested measures. However, the goals were generally formulated "sustainable boating and tourism" and very few indicators for monitoring how the measures are progressing towards the goals were suggested. Generally in the SAM plan the lesser developed ecosystem aspect in the System Description, the more general formulation of goals.

Integration (Score Medium)

Biodiversity (Score Medium): Even though there was a focus on marine biotopes in the System Description the Goals were not addressing this level of biodiversity, but rather the species level. However the terrestrial environment goals were formulated on biotope level. The measure phase of the plan was not as detailed as the description and goals concerning biodiversity. The main measures directly addressing biotopes and species were protection of areas and sustainable management of grasslands and forests, as well as hunting of some key predators (seals, mink and cormorant). The monitoring system was related to the detailed and often quantitative goals and hence matching the description and goals phases. Noteworthy is that even if invasive species was identified as one of the most severe threats to the PVs, there was no suggested monitoring. In summary there was matching System Description, Goals and Monitoring/Evaluation phases in terms of specificity and content whereas the Strategies/Measures were much more generally formulated and creating a gap in integration across management phases regarding ecosystem aspect Biodiversity.

Relations and Ecological Processes (Score Medium): The Goals concerning system relations were implicitly found in some of the biodiversity goals, e.g. the use of indicator species for certain biotopes. Since just some relations were addressed in the Goals there was not a complete match between the content and specificity in System Description and corresponding goals regarding system relations. For ecological processes the Goals management phase were more general and relative. The suggestions in Strategies/Measures in the plan did not match the degree of detail in the description concerning Relations and Ecological Processes. The relations of most concern were those of fish population dynamic, where limitation of predators in the food webs and enhancement of migration were suggested measures. The ecological process in focus was nutrient cycling and hence suggested nutrient retention strategies. For terrestrial environments (grasslands and forests) the degree of detail in the description was not captured in the following phases where suggested measures were enhanced management to limit overgrowth of grasslands and change in forest management schemes to increase the occurrence of old trees and dead wood. The monitoring function was weakly described and not addressing all aspects highlighted in the other phases, e.g. migration was not mentioned and nutrient cycling was generally addressed as “good water quality”.

Changes and Uncertainty (Score Low): The trends and prognosis formulated in the System Description were not captured in the following plan phases. Even though an overall adaptive approach was highlighted in the introduction of the plan, as well as the necessity of evaluation, this was not further specified and a monitoring program was not presented. There were no reported intentions to update the plan, rather monitoring towards the present goals. Climate change was mentioned and included as one IF in the System Description, but not further addressed. Uncertainty was addressed in terms of a general lack of knowledge expressed in the System Description, which was followed by Goals and Strategies/Measures including aspects of knowledge generation, information and raise of awareness. However, there were no suggested indicators of the outcomes of these particular measures. In summary there are severe gaps between the plan phases regarding both content and specificity of Changes and Uncertainty.

Scales (Score Low): Generally scales were only addressed in the System Description and to a very limited degree in the other phases. The targeted fish reproduction sites were both spatially and temporally explicit, but this was an exception among the Goals in the plan. The measures were temporally explicit only in an administrative sense, and only the suggestions of protected areas were somewhat spatially related. The maps in the description were not used to further specify the suggested measures. Some indicators involved spatial coverage of species communities.

Anthropogenic Processes (Score Medium): The Goals concerning Anthropogenic Processes very general, e.g. “sustainable fishing and boating” and some were prohibiting, e.g. “no dredging”. The Strategies/Measures management phase was most rigorous and detailed for this aspect of the system, where most suggested activities were about limiting the IFs by planning, knowledge generation, education and information. There were no indicators for monitoring related to Anthropogenic Processes, since the presented indicators focused on the end result, not the outcomes of a certain activity.

Supplementary Material S5

Score tables of the EBM assessment in in all five case study areas (A-E in Fig 2.) a) Scoring of the specificity in each combination of ecosystem aspect and plan phase (white cells), b) Average specificity score per ecosystem aspect across management phases (blue cells), c) Scoring of integration per ecosystem aspect (green cells), d) Aggregated score of systems thinking (orange cells). N/A indicates that this aspect was not reported in data.

<table>
<tr><th>HK area (A)</th><th colspan="4">a) SPECIFICITY</th><th></th><th></th><th></th></tr>
<tr><th>Management phases / Ecosystem aspects</th><th>System Description</th><th>Goals</th><th>Strategies/ Measures</th><th>Monitoring/ Evaluation</th><th>b) SPECIFICITY</th><th>c) INTEGRATION</th><th>d) SYSTEM THINKING</th></tr>
<tr><td>Biodiversity</td><td>high</td><td>medium +</td><td>low +</td><td>high</td><td>medium +</td><td>medium</td><td rowspan="5">MEDIUM</td></tr>
<tr><td>Relations and Ecological Processes</td><td>medium +</td><td>N/A</td><td>low</td><td>high</td><td>medium -</td><td>medium</td></tr>
<tr><td>Changes and Uncertainty</td><td>medium</td><td>medium</td><td>medium</td><td>medium +</td><td>medium</td><td>medium</td></tr>
<tr><td>Scales</td><td>medium</td><td>low</td><td>medium</td><td>medium -</td><td>medium -</td><td>low</td></tr>
<tr><td>Anthropogenic Processes</td><td>low</td><td>medium</td><td>high</td><td>medium</td><td>medium</td><td>medium</td></tr>
<tr><td></td><td></td><td></td><td></td><td></td><td>MEDIUM (total average score)</td><td>MEDIUM - (average score)</td><td></td></tr>
</table>

<table>
<tr><th>SNS area (B)</th><th colspan="4">a) SPECIFICITY</th><th></th><th></th><th></th></tr>
<tr><th>Management phases / Ecosystem aspects</th><th>System Description</th><th>Goals</th><th>Strategies/ Measures</th><th>Monitoring/ Evaluation</th><th>b) SPECIFICITY</th><th>c) INTEGRATION</th><th>d) SYSTEM THINKING</th></tr>
<tr><td>Biodiversity</td><td>high</td><td>medium</td><td>medium</td><td>medium</td><td>medium +</td><td>low</td><td rowspan="5">MEDIUM</td></tr>
<tr><td>Relations and Ecological Processes</td><td>medium</td><td>low</td><td>low</td><td>N/A</td><td>low</td><td>low</td></tr>
<tr><td>Changes and Uncertainty</td><td>low</td><td>low</td><td>low</td><td>low</td><td>low</td><td>medium</td></tr>
<tr><td>Scales</td><td>low</td><td>N/A</td><td>N/A</td><td>N/A</td><td>low -</td><td>low</td></tr>
<tr><td>Anthropogenic Processes</td><td>high</td><td>N/A</td><td>N/A</td><td>N/A</td><td>low</td><td>N/A</td></tr>
<tr><td></td><td></td><td></td><td></td><td></td><td>LOW + (total average score)</td><td>LOW (average score)</td><td></td></tr>
</table>

<table>
<tr><th>SAM area (C)</th><th colspan="4">a) SPECIFICITY</th><th></th><th></th><th></th></tr>
<tr><th></th><th>System</th><th>Goals</th><th>Strategies/</th><th>Monitoring/</th><th>b)</th><th>c)</th><th>d)</th></tr>
</table>

Management phases / Ecosystem aspects	Description		Measures	Evaluation	SPECIFICITY	INTEGRATION	SYSTEM THINKING
**Biodiversity**	high	high	medium	high	high	medium	MEDIUM
**Relations and Ecological Processes**	medium	medium	medium	medium	medium	medium	
**Changes and Uncertainty**	medium	low	medium	low	medium -	low	
**Scales**	medium	N/A	medium	N/A	medium -	low	
**Anthropogenic Processes**	high	low	high	medium	medium +	medium	
					MEDIUM (total average score)	MEDIUM - (average score)	

BA area (D)

Management phases / Ecosystem aspects	a) SPECIFICITY				b) SPECIFICITY	c) INTEGRATION	d) SYSTEM THINKING
	**System Description**	**Goals**	**Strategies/ Measures**	**Monitoring/ Evaluation**			
**Biodiversity**	high	medium +	high -	medium -	medium +	low	HIGH
**Relations and Ecological Processes**	high	low	low	N/A	low +	low	
**Changes and Uncertainty**	medium	medium	medium	N/A	medium -	low	
**Scales**	medium	low	low	low	low +	low	
**Anthropogenic Processes**	high	high	high	low	high -	medium	
					MEDIUM (total average score)	LOW (average score)	

NB area (E)

Management phases / Ecosystem aspects	a) SPECIFICITY				b) SPECIFICITY	c) INTEGRATION	d) SYSTEM THINKING
	**System Description**	**Goals**	**Strategies/ Measures**	**Monitoring/ Evaluation**			
**Biodiversity**	medium	medium	medium	high	medium +	medium	HIGH
**Relations and Ecological Processes**	medium	low	low	low	low +	medium	
**Changes and Uncertainty**	high	medium	low	medium	medium	medium	
**Scales**	medium	N/A	low	low	low	medium	
**Anthropogenic Processes**	high	medium	high	medium	high -	high	
					MEDIUM (total average score)	MEDIUM + (average score)	
